# Mushroom Quality Related with Various Substrates’ Bioaccumulation and Translocation of Heavy Metals

**DOI:** 10.3390/jof8010042

**Published:** 2021-12-31

**Authors:** Siti Maryam Salamah Ab Rhaman, Laila Naher, Shafiquzzaman Siddiquee

**Affiliations:** 1 Faculty of Agro-Based Industry, Jeli Campus, Universiti Malaysia Kelantan, Jeli 17600, Malaysia; maryam.f19d0055f@siswa.umk.edu.my; 2 Institute of Food Security and Sustainable Agriculture, Jeli Campus, Universiti Malaysia Kelantan, Jeli 17600, Malaysia; 3 Institute of Research and Poverty Management (InsPek), Jeli Campus, Universiti Malaysia Kelantan, Jeli 17600, Malaysia; 4 Biotechnology Research Institute, Universiti Malaysia Sabah, Jalan Universiti Malaysia Sabah, Kota Kinabalu 88400, Malaysia

**Keywords:** agriculture biomass, edible fungi, contamination, health, nutrition

## Abstract

Mushrooms are popular due to the nutrition contents in the fruit bodies and are relatively easy to cultivate. Mushrooms from the white-rot fungi group can be cultivated on agricultural biomass such as sawdust, paddy straw, wheat straw, oil palm frond, oil palm empty fruit bunches, oil palm bark, corn silage, corn cobs, banana leaves, coconut husk, pineapple peel, pineapple leaves, cotton stalk, sugarcane bagasse and various other agricultural biomass. Mushrooms are exceptional decomposers that play important roles in the food web to balance the ecosystems. They can uptake various minerals, including essential and non-essential minerals provided by the substrates. However, the agricultural biomass used for mushroom cultivation is sometimes polluted by heavy metals because of the increased anthropogenic activities occurring in line with urbanisation. Due to their role in mycoremediation, the mushrooms also absorb pollutants from the substrates into their fruit bodies. This article reviews the sources of agricultural biomass for mushroom cultivation that could track how the environmental heavy metals are accumulated and translocated into mushroom fruit bodies. This review also discusses the possible health risks from prolonged uptakes of heavy metal-contaminated mushrooms to highlight the importance of early contaminants’ detection for food security.

## 1. Introduction

Mushrooms are believed to have first emerged more than a million years ago. The oldest fossil of a gilled mushroom (*Gondwanagaricites magnificus*) confirmed its presence in Gondwana approximately 14 to 21 million years ago, which was during the Early Cretaceous [1]. People claimed that mushrooms were rare and supernatural species that could cure illnesses in ancient times. They also thought that the mushrooms consisted of healing qualities that could benefit humans. The Egyptians assumed that mushrooms were immortality plants and regarded them as “gifts from the Gods” [2], whereas the Romans posited that mushrooms were medicine from the gods that could assist in the hunt for missing objects and souls [2]. Mushrooms have long been recognised, particularly during the Chinese, Roman and Greek civilisations, where they consumed the edible mushrooms as high nutritional property foods [3]. Other than being highly nutritious, mushrooms are also famous due to the flavour and the texture of the flesh, which can enhance the aroma when cooked [4]. Mushrooms can also be utilised for different purposes, especially for sustainable human lifestyle [2] such as human food, animal feed, beverages, pharmaceutical, nutraceutical, fashion, architecture, packaging and filtration technologies [5].

Edible mushrooms can be wildly grown in the forests, especially on the dead and decaying matter such as a stump, rotten bark and soil [6]. Mushrooms can also be cultivated domestically or commercially. Mushrooms are one of the uppermost valuable commodity crops listed in Malaysia’s National Agro-Food Policy (2011–2020) [7]. Despite that more than 200 species of mushrooms have been used for functional foods worldwide for a long time, only about 35 species have been commercially cultivated [8,9]. The most commonly grown mushrooms in the world are *Agaricus bisporus* (button mushroom), *Auricularia auricula* (wood ear mushroom), *Flammulina velutipes* (winter mushroom), *Lentinula edodes* (shiitake), *Pleurotus* spp. (oyster mushrooms) and *Volvariella volvacea* (straw mushroom) [6]. There are mushrooms collected in the wild such as *Ganoderma* spp. (reishi mushroom), *Polyporus* spp. (porous ping-pong bat) and *Termitomyces* spp. (termite mushroom) [3]. 

The growth substrate is one of the factors that can highly affect the quality of edible mushrooms [10]. Given the saprophytic characteristic, the mushrooms obtain their nutrients by absorbing the dissolved organic matter from the deadwood and other decay materials. Nevertheless, Demkova et al. [11] reported that mushrooms have the ability to accumulate heavy metals in a large concentration, such as mercury (Hg), lead (Pb) [12,13], arsenic (As) [14,15], cadmium (Cd) [13,14,15], manganese (Mn), copper (Cu), iron (Fe) and zinc (Zn). Although some of the heavy metals such as Zn, Fe, Mn and Cu are essential metals in mushroom fruit bodies, others, such as Hg, Pb, As and Cd elements, are health hazards [16]. Furthermore, most of the elements can be bio-accumulated by the mushrooms, especially from the soil and substrates [17]. Therefore, this review discusses and summarises the accumulation of heavy metals by mushrooms cultivated on various substrates. The alternative substrates that have a permissible heavy metal content in the substrates for a better quality of edible mushrooms were also identified. 

## 2. Agricultural Biomass as Mushroom Cultivation Substrates

Several crops are cultivated and harvested around the world, with sugarcane (21%), corn (13%), rice (9%) and wheat (8%) being the four individual crops accounting for half of the global production of primary crops in the year 2018, whereas potatoes and soybean accounted for 4% of the world’s crop production [18]. Corn, rice and wheat are the crops that supply more than 42% of calories for the whole human population [19]. These crops are employed in manufacturing processes as raw materials and generate solid wastes such as rice straw, rice husk, wheat straw, corn silage, corn cob and sugarcane bagasse. Table 1 below shows the agricultural biomass produced in selected countries from each continent.

In Malaysia, the agricultural sector has become one of the country’s major contributors to its economy. As the agriculture sector expands, crop yields have risen, thereby increasing the generation of agricultural waste such as paddy straw, coconut husk, corn silage, sugarcane bagasse and others. According to Tambichik et al. [42], the amount of waste generated by agricultural and construction industries grew annually, particularly in Malaysia. However, Malaysia has disposed of 1.2 million tonnes of agricultural waste into landfills yearly [43]. Large amounts of unused lignocellulosic by-products are available in tropical and subtropical areas, especially in Malaysia [44]. Poor management of agricultural waste can lead to environmental pollution. For instance, one of the most important issues that might pollute the environment is the open burning of paddy straw at the paddy field. Besides, the waste from the pineapple leaves and pineapple skins are usually left rotten on the farm. Therefore, each individual needs to be made aware of the importance of taking care of the environment. To reduce the environmental pollution caused by inefficient agro-waste management, the waste produced can be utilised as a substrate for mushroom cultivation [45]. Table 2 shows the list of various agricultural residues used for mushroom cultivation from previous research.

Un-utilised agriculture waste increases year by year if proper waste management is lacking to overcome the problems. Several types of agricultural waste are freely available in the world. Agriculture waste mainly contains lignocellulose as the main component, and it is built with cellulose, hemicellulose and lignin, as well as the extractives and minerals [62]. These three major structural components are found in the entire parts of the vascular plants, serving as structural support systems. Basically, the lignin components in the biomass only cover 10% to 25% of the biomass on a dry basis, while the cellulose and hemicellulose typically range from 40% to 60% and 20% to 40% on a dry basis, respectively [63]. The major lignocellulose agricultural residues produced are paddy straw, wheat straw, barley straw, corn stover, sorghum stalks, coconut husk, sugarcane bagasse, oil palm wastes, pineapple skin and banana leaves [64]. Due to the inclusion of cellulose, hemicellulose and lignin in the agricultural biomass, it makes the residue difficult to break down. Nevertheless, the decomposition process of fungi can easily degrade agricultural biomass [49]. Fungi are natural decomposers that can degrade agricultural biomass and catalyse the decomposition process. Therefore, the involvement of fungi in re-utilising abundant and renewable resources such as agriculture waste or biomass is one of the alternative ways to solve the abundant agriculture waste produced in the field.

Mushrooms are the spore-bearing and the largest fungi [5] with the potential of degrading the lignocellulosic-agricultural residues into food because they lack the chemoheterotrophic extracellular digestion process. The lignocellulose product obtained from the decomposition process allows the mushroom to use it as a source of nourishment [45]. However, a few fermentation factors such as medium composition, the ratio of carbon to nitrogen, pH, temperature and air composition influence the obtained lignocellulose product [65]. Nutrient sources for mushrooms mostly come from the substrates, affecting the chemical, functional and sensorial characteristics of the mushroom fruit bodies [66]. 

Minerals are an integral part of the substrate necessary for mushrooms’ growth. The substrate can also be supplemented with minerals, which when applied in adequate amounts can improve the incubation and fructification speed. In fact, lignocellulosic substrate materials usually have a lower mineral content compared to others [65], and they need supplements to fulfil the requirements for cost-effective production. The addition of supplements to the substrates can increase the growth rate of the mushroom fruit bodies. As reported by Fanadzo et al. [67], the cultivation of *P. ostreatus* on the wheat straw with the application of cottonseed hull as supplements can increase mushroom biological efficiency (BE) compared to maize bran, which is 70.4% and 49.4%, respectively. It proved that the addition of different minerals such as supplements could help the fructification in some mushroom strains [68]. The application of supplements must be in a desirable amount that is suitable with the requirement of the mushroom species. Excessive application of supplements towards mushroom cultivation leads to increasing the mineral contents in the mushroom substrate that culminates into excessive bed temperature, the possibility of mycelium mortality [67] and undesirable flavour towards food and contamination [69]. A few trace elements can also be naturally found in the mushroom substrates, such as calcium, zinc, manganese, iron, copper and molybdenum cations. However, modernisation has heightened the demand for these trace elements in various industries, which has led to increased exploitation of raw materials. The activities will cause the mobilisation of a large amount of gas and contaminants in the ecosystems [70]. 

## 3. Sources of Heavy Metals in Mushroom Substrates

Heavy metals are a group of metals and metalloids that have relatively high density and high toxicity, even at permissible limits, such as Pb, As, Hg, Cd, Zn, Ag, Cu, Fe, Cr, Ni, Pd and Pt [71]. Heavy metals can also be classified as inorganic chemical hazards that are mostly found in contaminated areas [72], and the common examples include Pb, Cr, As, Zn, Cd, Cu, Hg and Ni [73]. Kabata-Pendias et al. [74] reported that heavy metals could occur naturally in the soil environment due to the weathering process of the parent material at a trace level of less than 1000 mg kg^−1^. The heavy metals can be eliminated and uptakes by the microorganisms depends on the chemistry of ion metals, cell wall compositions in the microorganisms, physiology of the cell and phytochemical factor such as pH, temperature, time ionic strength and metal concentration [75] The industrial revolution has facilitated advanced technology with broad effects on environmental pollution, especially in the industrial sector [76,77]. Rapid industrialisation and urbanisation in emerging countries have caused increased heavy metals’ contamination and pose a risk to human health and the environment [78].

Soil, water and air are the major components of the environment that are usually polluted by heavy metal contaminants produced by anthropogenic activities [79]. Moreover, heavy metals have also been introduced into agricultural systems through land application of sewage sludge, organic waste manure, industrial waste and irrigation with wastewater [80]. Heavy metal contamination has a wide range of effects on agriculture, from agricultural soil to the products consumed by humans. Given the growing usage of agrochemicals and inorganic fertilisers, modern agricultural practices have resulted in agricultural pollution that results in the ecosystem and environmental damage [81]. Inorganic fertiliser, which includes liming, irrigation and sewage sludge, are the significant sources of heavy metals in agricultural soil (Table 3). Different anthropogenic activities can influence heavy metal concentrations. The concentration of heavy metals differs among anthropogenic activities due to the application of phosphate, potash and nitrate fertiliser and lime.

**Figure 1:**
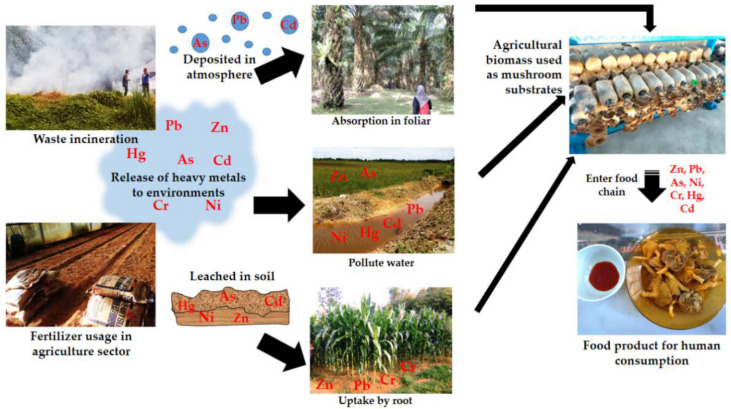
Bioaccumulation of heavy metals in mushrooms enters the food chain.

Fertiliser is important in preserving plant growth. Plants need essential macronutrients such as N, P, K, S, Ca and Mg for their growth. However, the soil needs to be supported with additional supplements due to the deficiency of a few heavy metals, such as Fe, Zn, Cu, Co, Mn, Mo and Ni [92]. Cereal crops cultivated on Cu-deficient soil are occasionally treated with Cu as a soil amendment [72]. Other than fertiliser application, pesticides are also one of the agriculture essentials which include herbicides, insecticides and fungicides. The function of pesticides is to control the pests and weeds that are detrimental to the crops. Pesticides used in agriculture usually contain metals. For example, lead arsenate is used in fruit orchards for parasite control [72]. In Malaysia, the use of herbicides covered about 83% of the total pesticides used in agriculture [62]. The application of phosphate fertiliser, inorganic fertilisers and pesticides in the plantation have contributed to the varying concentration of Zn, Pb, Cr, Cd and As in the soil [72,84,85]. For instance, leachate of Cd in the soils will be taken up by the plants and highly deposited on leaves that may be consumed by animals or humans [88] (Figure 1). 

Apart from fertilisers and pesticides, manure and bio-solids also add Cu, Zn and Mn into the soil, while sewage sludge contributes Cu, Zn, Mn, Cr, Pb, Ni and Cd [80]. Growers also use animal manure from poultry, cattle and pigs for their crops. Although the application of animal manure could be considered as organic fertiliser, the excessive application to the soil or long-term application of manure might increase soil salinisation and intensify the accumulation of heavy metals in plants [93,94]. The concentration of Cu, Mn, Zn and Cr was demonstrated to be higher in plant shoots that were treated with pig slurries [89]. The animal manures contain high concentrations of heavy metals such as As, Cu and Zn, and hence the long-term application on soil can increase the concentration of these heavy metals. Bio-solid, also known as sewage sludge, is an organic solid product that is mostly produced during the wastewater treatment process. The organic solid can be recycled and usually applied in agriculture as fertiliser [72]. Thus, the leachate from the applied bio-solid can also be incorporated into the soil and increase the concentration of the heavy metals [95].

Furthermore, the heavy metal contamination in the soil is caused by atmospheric deposition through mining, transportation and waste incineration. Metals are released into the air usually through fugitive emission, emission of air, gas or vapour, however, some heavy metals such as As, Cd and Pb have volatile characteristics when reacting with high temperatures. These heavy metals are very toxic when inhaled by humans and may cause serious illness. The contaminated smoke contains solid particles that are deposited on land or sea. Exposure to heavy metals from the air is more dangerous because the contaminants can widely spread and increase the deposited area. 

Besides, industrial activities also contribute to heavy metal contamination. The levels of heavy metals in soil can increase due to mining activities by accelerating the bedrock weathering [96]. For instance, gold mining contributes to the Hg in the environment [72], while As, Cd and Fe are produced by the coal mines, which causes serious problems in the soil and negatively affects human health. Other than that, the metals will be distributed into the water and surface sediments, resulting in contamination of water sources and the land that are used as plant nutrition sources [96]. Therefore, the plants directly uptake the heavy metals from mining activities, which are then translocated into the food chain. The contaminations are stated as a longstanding compound and can cause long-term health problems [97].

Besides the natural existence of metals in soil, most human activities also contribute towards the contamination of the soil by heavy metals. Due to the excessive contaminants deposited into the soil, the contaminants will also be deposited into the plants due to the phytoremediation process occurring from the soil through the roots [98]. The plants will absorb the heavy metals from the soil and release the natural substances to supply the nutrient through the roots. However, the heavy metals will remain in the plants.

## 4. Bioaccumulation of Heavy Metals in Mushrooms

Heavy metal contamination of the rivers, agricultural areas and lands has been reported [99]. The heavy metals can be eliminated from the contaminated area through several methods, such as chemical precipitation, coagulation with alum or iron salts, membrane filtration, reverse osmosis, ion exchange and adsorption [100,101]. The biosorption method is a promising low-cost metal removal method, where microorganisms are used to eliminate the metals [102]. In this context, the heavy metals can be eliminated and taken up by the microorganisms depending on the chemistry of ion metals, microorganisms’ cell wall compositions, physiology of the cell and phytochemical factors such as pH, temperature, time ionic strength and metal concentration [102]. 

Crops, vegetables and fruits are stated to have low heavy metal concentrations compared to the levels in mushroom fruit bodies [103]. Mushrooms can easily absorb heavy metals from the environment due to the ability to undergo the mycoremediation process compared to other plants that grow in the same area [104]. Additionally, mushrooms can absorb either essential or non-essential metals. Essential metals are those that are required at certain levels by humans for biological systems, such as Fe, Cu, Mn and Zn. However, excessive levels of these essential metals can also cause negative effects on the organisms. Likewise, non-essential metals such as Cd, Pb and As are toxic and can cause serious illness to the organisms [105]. The accumulation of heavy metals in mushroom fruit bodies is noted to be affected by mushroom species, substrates’ composition and bioavailability of the metals [106]. 

Heavy metal accumulation varies based on different mushroom species. *Pleurotus* sp. is a mushroom species that has a very high potential in the biosorption of environmental contaminants (Table 4). Specifically, *P. sajor-caju*, *P. ostreatus* and *P. florida* were reported to absorb Cd, Cu, Ni, Fe, Zn, Mn, Hg and Pb [71,103,107,108]. *Pleurotus* sp. are widely grown all over the world, especially in the forest [104]. The highest heavy metal recorded in *Pleurotus* sp. is Fe at 243.92 mg/kg of dry weight [103], while *Lentinula edodes* recorded the highest amount of As compared to other mushroom species, which was 1.21 mg/kg of dry weight, exceeding the permissible limit by the WHO 2015 (Table 5). The bioaccumulation potential in each of the mushroom species shows the mushroom’s capability to absorb the metals based on a species-to-species mechanism [102]. 

Heavy metals in mushrooms also arise from their growth environment, especially from their growth substrates. Other than wild-growing mushrooms, growers often use agriculture biomass to cultivate the mushrooms. Nevertheless, some of the agriculture biomass contained heavy metals due to anthropogenic activities (Table 3). The heavy metals may remain in the biomass and be transported into mushroom fruit bodies through cultivation. The uptake of metals from the biosorption process by the mushrooms is unevenly distributed within the mushroom fruit bodies. Mushrooms contain abundant hyphae in the mycelium that help the biosorption of nutritive elements and heavy metals in the substrates [104]. The hyphae have a rough texture and can attract metals and other chemical contaminants [102]. The mycelia of the mushroom can also act as a biological filter and possible sorbent to the mushroom fruit bodies. Fe, Hg, As, Cd, Zn and Pb have been detected in high concentrations in the sporocarps of the wild mushrooms compared to the stipe [15]. The cell wall of this mushroom species helps in the biosorption of heavy metals into the fruit bodies, and they have been reported to have carboxylic, amino, thiol, phosphate and hydroxide groups computed in the cell wall [109]. 

## 5. Translocation of Heavy Metal Contamination in Mushroom Food Chain

Mushrooms are popular due to their nutritional value, which offers several benefits to consumers. Due to the ability to undergo the mycoremediation process, the mushrooms can absorb heavy metals, especially the wild mushroom that grows in contaminated areas [104]. However, the main concern is the consequences that come from heavy metal mitigation in the trophic chain, comprising the soil, plants, animals and humans [115]. The permanent entry of heavy metals into the food chain can be extremely dangerous, especially for human health. Heavy metals can mainly spread in humans and animals through food, air or skin [116]. Some edible mushrooms can accumulate high concentrations of certain metals, such as As, Cd, Cu, Hg and Pb [117]. Most of these metals contain major toxic and harmful effects on human health at low levels. Hence, little effort has been made to investigate the potential danger of mushroom consumption to human health. 

Arsenic (As) is one of the metals that can be found in marine organisms such as fish, squid, clams and other marine invertebrates [118], as well as in mushroom fruit bodies and the environment [119]. The permissible As level in food and vegetables according to FAO/WHO (2015) is at 0.2 mg/L (Table 4). Prolonged consumption of high arsenic content food may cause cancer and skin lesions [120] and could be highly toxic when in an inorganic state. *Lentinula edodes* is reported to mainly contain inorganic As, which is 84% (1.38 mg/L) and above the permissible As safe consumption level [121]. Additionally, the ingestion of As at a dangerous level can lead to liver disease, coma and death in the worst-case scenario [122]. 

Another heavy metal in the mushroom food chain that is detrimental to humans is Cd. Exposure to and consuming high Cd level food remains a high risk for the deterioration of human health. Chunhabundit [123] reported that exposure to Cd could lead to chronic kidney disease, osteoporosis, diabetes, cardiovascular disease and cancer. Entrance of Cd into body systems can cause renal damage by proximal tubule dysfunction. Exposure to Cd can induce oxidative stress and modification of DNA expression [124]. 

Pb has also been detected in *Agaricus bisporus* collected from urban areas, and the Pb content in *A. bisporus* was found to be 0.54 mg/kg dry weight [12]. Environmental scientists reported that Pb toxicity is dangerous to plants, animals and humans [125]. Several diseases could develop when Pb enters and contaminates the food chain. Pb can also be found in straw mushroom fruit bodies that grow on Pb-contaminated rice straw and stubble, where the highest Pb concentration is recorded in egg and the mature stage of straw mushroom (*V. volvacea*) [126]. The chances of these metals in straw mushrooms entering the consumer food chain are high since the button to egg stages are the preferred harvesting stages for straw mushrooms [127]. By consuming high Pb content food, the antioxidants’ cell defence system may be disrupted, leading to cell death [125]. The metals can also enter the central nervous system and cause behavioural and developmental disorders, such as learning disabilities, attention deficit disorders, brain damage, muscle weakness, anaemia and renal dysfunction [119]. The most dangerous effects can also occur following long-term exposure to Pb contamination. 

Apart from the metals, Cu is an essential mineral that is beneficial to various physiologic and metabolic effects in humans. Cu is an important component for bone formation and metabolism of iron and heme synthesis in the human body and enhancing the proper function of the nervous system [128]. However, excessive intake of Cu might lead to Cu toxicity and is a potential carcinogen [129]. The carcinogen in mushrooms can promote the growth of tumours in humans. Furthermore, prolonged consumption of Cu-contaminated food can cause various types of health problems, such as hepatic cirrhosis, anaemia, osteoporosis, cell hemolysis and kidney disorders [130,131,132]. The accumulation of Cd has been reported in *Pleurotus* species that grew on metal-enriched duckweed and the accumulation of Cd content was above permissible limits [133].

Mercury (Hg) contamination is one of the most important issues, especially in the environment and humans [133]. Hg exposure has become a major public health concern worldwide [134]. The concentration of Hg in edible mushrooms is extreme, especially for those cultivated in a contaminated environment. The issue of Hg contamination in food has become a concern if it gains entry into the human body. Slavik et al. [135] found high Hg levels in edible mushrooms cultivated in highly contaminated areas in Spis. Therefore, it is not advisable to consume mushrooms grown in high-risk areas because Hg can cause nervous system damage in adults and impaired neurological development in infants and children [136]. Consistent exposure to Hg can also induce oxidative stress and mitochondrial dysfunction [137]. 

Mushroom intake was defined as any amount of mushroom consumed based on USDA food codes including the foods that were mixed with mushrooms [138]. The standard mushroom daily intake is 84 g, which is about three ounces per person [139]. In Malaysia, it is estimated that the rate of mushroom consumption is 2.4 kg per person in the year 2020 [140]. High consumption of contaminated mushrooms can lead to high exposure to heavy metals. The most crucial factor to reduce the risk of contamination of heavy metals for human health is by monitoring the sources of the mushrooms. The risk of exposure towards heavy metals from the consumption of mushrooms can be determined by estimated daily intake, target hazard quotient, carcinogenic risk and the hazard index [52], which can be calculated according to the following equation [141]:

(1) $$\mathrm{EDI}\;\left(\frac{\mathrm{mg}}{\mathrm{kg}.\mathrm{day}}\right)=\frac{C\;\times \mathrm{IR}\;\times \mathrm{ED}\;\times \mathrm{EF}}{\mathrm{BW}\;\times \mathrm{AT}}$$

where EDI represents estimated daily intake, C is concentration of heavy metal in the edible mushroom (mg/kg), IR is ingestion rate (kg/person/daily), EF is exposure frequency (350 day/year), ED is exposure duration (children = 6 years, adults = 30 years), BW is body weight (children = 15 kg, adults = 70 kg) and AT_n_ (EF × ED) is average time if exposure (children = 2190 days, adults = 10,950 days).

The target hazard quotient (THQ) can be expressed as the ratio of EDI to the reference dose (RfD), which can be calculated using the following equation:

(2) $$\mathrm{THQ}=\frac{\mathrm{EDI}\;\left(\mathrm{mg}/\mathrm{kg}-\mathrm{day}\right)}{\mathrm{RfD}\;\left(\mathrm{mg}/\mathrm{kg}-\mathrm{day}\right)}$$

The RfD value can be determined as 4.0 × 10^−^^2^, 7.0 × 10^−^^1^, 3.0 × 10^−^^1^, 3.5 × 10^−3^, 1.5, 1.0 × 10^−4^ and 1.4 × 10^−^^2^, 2.0 × 10^−^^2^ and 1.0 × 10^−3^ mg kg^−^^1^ day^−^^1^ for Cu, Fe, Zn, Pb, Cr, Mn, Ni and Cd, respectively [141]. However, the THQ value differs based on various heavy metals’ concentrations in edible mushrooms, duration of exposure, rate of ingestion, type of heavy metals and number of heavy metals analysis [141].

## 6. Formulation of Mushroom Substrate and Heavy Metal Relation

Each mushroom species needs optimal nutrition in the cultivation substrates that help the mushroom growers achieve the highest yield in a short cultivation period. Mushrooms cultivated on a commercial scale require good-quality substrates that can give a high yield in return. Mushrooms, especially *Pleurotus* spp., need substrates containing carbon, nitrogen and inorganic compound for their growth [66]. The main mushroom substrates are usually low in nitrogen and high in carbon, where it contains cellulose, hemicellulose and lignin such as paddy straw, wheat straw, cottonseed hulls, sawdust, wastepaper, leaves and residue from the sugarcane [142]. Most of the growers used sawdust and wheat straw as the commercial mushroom substrates, supplemented with different commercial supplements such as rice bran, cottonseed hulls and other cereal bran [143]. 

However, most commercial substrates, especially the rubber sawdust and wheat straw, have been polluted with different kinds of pollutants and caused an increased accumulation of heavy metals in the substrates (Table 6). Given the ability to absorb heavy metal ions, sawdust has a high content of heavy metals compared to wheat straw [144]. Moreover, the heavy metals in *Pleurotus* sp. fruit bodies can be affected by the growth substrates [145]. Heavy metals are non-biodegradable by any biological and physical process and could remain in the soil for years. Once the heavy metal is leached, it will be immobilised in the soil and leach into groundwater and might be accumulated and taken up by the plants [145]. An increased concentration of heavy metals and inorganic matter in the plants will also reduce the pH, thereby facilitating easier detection of the biological materials in the plants [103]. Furthermore, high heavy metal content in plants can affect the content in the mushroom because the fruit bodies are able to accumulate the heavy metal that leached into the plants. Thus, it is important to choose the alternative mushroom substrates with a heavy metal content at the permissible level.

## 7. Conclusions

There are high amounts of nutrients in most agricultural biomass, and the raw materials are particularly suitable for growth substrates. The utilisation of agricultural biomass or wastes as sources of mushroom substrates offers suitable ways to overcome the excess waste in the agriculture field. The occurrence of essential and non-essential elements in agricultural biomass such as mushroom substrates indicates the quality of the production of mushroom fruit bodies. Good-quality mushrooms uptake nutrients from good-quality substrates. Unfortunately, the non-essential elements can be translocated into the mushroom fruit bodies through the mycoremediation process performed by the edible mushroom. The contaminants that leached into the substrates could enter the human food chain through consumption of the mushroom fruit bodies, leading to various health problems either in short or long consumption periods. Therefore, to improve future national food security, detection and selection of suitable agricultural biomass as mushroom substrates is crucial to avoid the bioaccumulation and translocation of heavy metals into the food chain.

## Figures and Tables

**Table 1:** Agricultural biomass produced in selected countries of the continents.

Continents	Country	Agricultural Biomass Produced	References
Asia	Philippines	Rice hulls, rice straw, corn cobs, corn husks, corn leaves, corn stalks, coconut husk, sugarcane leaves, sugarcane bagasse, banana trunk, banana leaves, banana peels, pineapple crown, pineapple peel, coffee pulp, mango peel, mango pulp, cacao pods, cassava trunk, cassava leaves, cassava peel, peanut pods.	Cruz, 1997 [20]
	Vietnam	Rice straw, rice husk, corn leaves, corn cobs, cassava leaves and tops, cassava stalks, pulp and cortex, sugarcane leaves and bagasse, coffee stem, leaves, husk and coffee ground, Soybean steam, leaves, branches and shells.	Son et al., 2021 [21]
	China	Rice husk and straw, corn cobs, husk, leaves and stalks, sugarcane bagasse, wheat straw	Atinkut et al., 2020 [22] Millati et al., 2019 [23] Basalan et al., 1995 [24] USDA, 2018 [25] Birru et al., 2016 [26] Bakker et al., 2013 [27]
	Thailand	Rice straw and husk, oil palm empty fruit bunch, sugarcane bagasse, stumps and leaves, coconut husk and shells, cassava leaves and peels, rubber sawdust	Papong et al., 2004 [28]
	Malaysia	Rice straw and husk, oil palm empty fruit bunch, oil palm trunk and frond, timber, coconut husk, coir and trunk, sugarcane bagasse, stumps and leaves	Siddiqui et al., 2019 [29] Ozturk et al., 2017 [30]
	Indonesia	Rice husk and straw, corn cob, stalk and husk, cassava stalk, oil palm mesocarp fibre, oil palm kernel shell and empty fruit bunch, coconut husk and shell, forest and wood residues, sugarcane tops and bagasse Coffee leaves, pulp, husk and spent coffee grounds	Budhijanto et al., 2019 [31] Pranoto et al., 2013 [32] Abdul Wahid et al., 2017 [33] Klingel et al., 2020 [34]
	India	Rice husk and bran, Wheat bran and straw, corn stover, husk and skins, miller stover, sugarcane tops, bagasse and molasses Apple pomace	Phonbumrung et al., 1998 [35] Arvanitoyannis et al., 2008 [36] Bhuvaneshwari et al., 2019 [37] Lyu et al., 2020 [38]
Europe	Covers 28 EU countries	Vine shoots, grape stalks and grape pomace Coffee leaves, pulp, husk and spent coffee grounds Apple pomace	Pardo-Gimenez et al., 2007 [39] Klingel et al., 2020 [34] Lyu et al., 2020 [38]
Africa	Tanzania	Rice husks, coconut shells, cashew nuts shells and palm fruit shells	Mdoe, 2014 [40]
Oceania	Australia	Coffee husk and coffee pulp powder Apple pomace	Bio Bag, 2020 [41] Lyu et al., 2020 [38]

**Table 2:** Various agricultural residues used for mushroom cultivation.

Mushroom Species	Agriculture Residue as Substrates	References
*Pleurotus* sp. (oyster mushroom)	Wheat straw, rice straw, soybean straw, corn straw, peanut straw, rape straw. Sawdust, corn husk, corn cob, corn stalk. Date palm leaves, wheat straw, sawdust. Rice straw, wheat straw, cotton straw, tea leaves, banana leaves. Cotton waste, sawdust Paddy straw, coir pith, banana leaf Wheat straw and olive mill waste Vineyard pruning and grape pomace	Wu et al., 2019 [46] et al., 2015 [47] Alananbeh et al., 2014 [48] Kamthan et al., 2017 [49] Odunmbaku et al., 2018 [50] Udayasimha et al., 2012 [51] Ruiz-Rodriguez et al., 2010 [52] Sanchez et al., 2002 [53]
*Lentinula edodes* (button mushroom)	Rice bran, coffee pulp, coffee husk, spent coffee grounds, sugarcane bagasse, corn cob, millet straw, wheat straw, tea leaves, peanut hulls, cottonseed hulls, sunflower seed hulls, dried grass powder, water hyacinth, etc. Wheat straw Wheat straw, corn cobs, oak-wood sawdust Beech sawdust, wheat bran, olive oil press cakes, gypsumGrape stalks and vine shoots	Kamthan et al., 2017 [49] Mata et al., 2018 [54] Philippoussis et al., 2007 [55] Gregori et al., 2012 [56] Pardo-Gimenez et al., 2007 [39]
*Schizophyllum commune* (split gills mushroom)	Banana leaves, coconut leaves, paddy straw, coir dust, rubber sawdust	Ediriweera et al., 2015 [57]
*Volvariella volvacea* (straw mushroom)	Tea leaves, paddy straw, water hyacinth, oil palm bunch, oil palm pericarp waste, banana leaves, sawdust, cotton waste and sugarcane bagasse. Paddy straw, cotton waste, banana leavesRice straw	Kamthan et al., 2017 [49] Mangunwardoyo et al., 2018 [58] Biswas et al., 2014 [59]
*Ganoderma lucidum* (lingzhi mushroom)	Sawdust Broad beanstalks, cotton stalk, corn straw, paddy straw, sugarcane bagasse and wheat straw	Kamthan et al., 2017 [49] Rashad et al., 2019 [60]
*Flammulina velutipes* (enoki mushroom)	Olive waste and poplar wood shavings	Rugolo et al., 2016 [61]

**Table 3:** Sources of heavy metals in agricultural soil.

Sources	Type of Activities	Heavy Metal	References
Fertilisers	Phosphate, Potash and Nitrate fertiliser Lime	Zn, Pb, Cr, Cd, As	Karalic et al., 2013 [82], Atafar et al., 2010 [83], Sun et al., 2013 [84], Kelepertzis 2014 [85].
Pesticides	Herbicides Insecticides Fungicides	As, Co, Cr, Ni, Pb, Cu, Zn, Cd	Defarge et al., 2018 [86], Kelepertzis, 2014 [85], Quinteros et al., 2017 [87], Srivastava et al., 2017 [88]
Manure and bio-solid	Livestock manure Composts Sewage sludge	Cu, Zn, Mn, Cr, Pb, Ni, Cd	Provolo et al., 2018 [89], Wuana et al., 2011 [72], Srivastava et al., 2017 [88], Sharma et al., 2017 [85]
Wastewater	Irrigation with municipal wastewater Industrial waste water	Zn, Cu, Ni, Pb, Cd, Cr, As, Hg	Wuana et al., 2011 [72] Balkhair et al., 2016 [90], Woldetsadik et al., 2017 [91]
Atmospheric deposition	Mining, transportation, waste incineration	Cr, Pb, Zn, As, Cd, Hg, Ni	Wuana et al., 2011 [72]

**Table 4:** Absorption of heavy metals in mushroom fruit bodies.

Mushroom Species	Edibility	Heavy Metal Concentration in Fruit Bodies	References
*Pleurotus sajor-caju*	Edible	Cd (98.94), Ni (97.22), Fe (88.24) *	Yadav et al., 2020 [71]
*Pleurotus ostreatus*	Edible	Cu (53.56), Fe (220.87), Zn (89.68), Mn (47.55) * Pb (3.24), Cd(1.18), Hg (0.42), Cu (13.6), Mn (6.27), Zn (29.8), Fe (86.1) *Pb (0.11), Cd (0.55), Hg (0.31), Fe (48.6), Cu (5.0), Mn (10.3), Zn (19.3) * Cd (11.2), Hg (1.2), Zn (0.8), Pb (0.0) *	Gebrelibanos et al., 2016 [103] Demirbas, 2001 [110] Tuzen et al., 1998 [107] Lasota et al., 1990 [108]
*Pleurotus florida*	Edible	Cu (53.56), Fe (243.92), Zn (95.26), Mn (41.29) * Cd (98.93), Ni (97.22), Fe (84.84) *	Gebrelibanos et al., 2016 [103] Yadav et al., 2020 [71]
*Pleurotus eryngii*	Edible	Cu (0.00), Zn (1.00), Cd (0.03), Co (1.00), Ni (0.00) ***	Drzewiecka et al., 2010 [111]
*Lentinula edodes*	Edible	Ni (1.6), Cr (3.25), Pb (0.84), Cd (1.15), As (1.21), Hg (0.0) *	Na et al., 2014 [75]
*Schizophyllum commune*	Edible	Hg (0.0), Fe (11.00), Zn (6.46), Pb (1.56), Cu (1.76), Cd (2.25), Ni (4.24) *	Udochukwu et al., 2014 [112]
*Volvariella volvacea*	Edible	Cu (93.59), Pb (98.69)Hg (0.0), Fe (8.25), Zn (27.33), Pb (1.25), Cu (1.55), Cd (4.88), Ni (5.75) * Fe (322.5), Cu (101.8), Zn (36.5), Mn (78.5), Cr (0.24), Pb (0.25) **	Yadav et al., 2013 [71] Udochukwu et al., 2014 [112] Mohiuddin et al., 2015 [113]
*Ganoderma lucidum*	Edible	Pb (0.08), Cd (0.11), As (0.03), Hg (0.01) * Fe (303.0), Cu (72.5), Zn (52.2), Mn (64.0), Cr (0.21), Pb (0.13) **	An et al., 2020 [114] Mohiuddin et al., 2015 [113]

* mg/kg dry weight, ** µg g^−1^ dry weight, *** mM kg^−1^ dry weight.

**Table 5:** Permissible level for heavy metals in food and vegetables according to FAO/WHO (2015).

Metals	WHO/FAO (mg/L)	Normal Range in Plant (mg/L)
Cu	30.0	2.5
Pb	2.0	0.50–30.0
Zn	60.0	20.0–100.0
Fe	48.0	400.0–500.0
As	0.2	0.2–1.5

**Table 6:** Chemical and heavy metal composition in commercial mushroom substrates.

Mushroom Species	Mushroom Substrates	Biochemical Composition	Substrate Heavy Metal Content	References
*Pleurotus sajor-caju* and *Pleurotus ostreatus* (grey oyster mushroom)	Rubber sawdust with rice bran and hydrated lime	Protein (14.5), Carbohydrate (61.45), Fat (23.22), Lignin (70.27), Ash (5.146) *	Cu (0.020), Zn (0.539), Mn (0.580), Fe (3.233) *	Abd Rasib et al., 2015 [146] Boamponsem et al., 2013 [145]
*Pleurotus florida* (white oyster mushroom)	wheat straw with 2% of aqueous formalin	Protein (16.1), Carbohydrate (63.57), Fat (23.78), Lignin (70.67), Ash (5.299) *	Cu (1.034), Fe (0.920), Zn (1.483), Mn (0.660) ***	Abd Rasib et al., 2015 [146] Gebrelibanos et al., 2016 [103]
*Pleurotus eryngii*(king oyster mushroom)	50% sawdust with 25% cotton seed hulls and 25% wheat bran supplemented with gypsum	Protein (21.5), Ash (6.02), Fiber (62.0), Lipid (0.54) *	As (0.16), Cr (0.12), Cd (0.83), Hg (0.00006), Pb (2.110), Na (244.00), K (3927.00), Ca (4671.00), Mg (1391.00), P (1262.00), Cu (2.90), Mn (13.10), Zn (5.60), Fe (19.50) **	Sun et al., 2013 [84]
*Ganoderma lucidium*(ganoderma)	Rubber sawdust with rice bran and hydrated lime	Protein (36.6), Carbohydrate (70.42), Fat (25.56), Lignin (72.13), Ash (5.605) *	Cu (24.00), Mn (31.00), Zn (31.00), Cd (<0.05), Hg (0.01), Pb (2.00) **	Abd Rasib et al., 2015 [146] Tham et al., 1999 [147]

* g/kg dry weight, ** µg/g dry weight, *** mg/kg dry weight.

## Data Availability

Not applicable.
